# Selfies and the (Creative) Self: A Diary Study

**DOI:** 10.3389/fpsyg.2017.00172

**Published:** 2017-02-08

**Authors:** Maciej Karwowski, Arkadiusz Brzeski

**Affiliations:** Department of Educational Sciences, The Maria Grzegorzewska UniversityWarsaw, Poland

**Keywords:** selfie, creativity, creative activity, diary study, multilevel modeling

## Abstract

In this diary investigation, over 2 weeks we monitored the intensity of selfie posting among 292 Facebook users (60% females), aged between 18 and 50, to estimate the extent of selfying’s day-to-day variability and its predictors. The obtained effect was large; 64% of the variability in selfying was located within rather than between individuals. Day-to-day changes in creative activity explained a significant proportion of selfying, similarly as previous creative achievement did. At the same time, intelligence was negatively linked to the intensity of selfie posting and moderated the relationship between creative achievements and selfying. We discuss hypothetical links between selfie posting and the situational and individual differences characteristics related to creativity and cognitive abilities.

## Introduction

Consider for a moment some seminal achievements in the history of art: the portrait of a man in red chalk, attributed to Leonardo da Vinci; a collection of Pablo Picasso’s self-portraits showing evolution of his artistic style; or a dozen of Vincent van Gogh’s self-portraits, with their famous mirror-like character. Now, let us switch to XXI century with its new technologies, smartphones, Facebook posts, and Twitter tweets. And here’s the point: would Leonardo, Picasso, or van Gogh be selfying instead of self-portraying today? Do selfies hold any creative value or should they be perceived exclusively as a proof of narcissism and vanity (Sorokowski et al., 2016)? These provocative questions inspire our endeavors presented in this article.

This paper explores the selfie phenomenon, but does not focus on Leonardo’s or Picasso’s selfies. Even if some links and regularities between artists’ self-portraits and naïve people’s selfies were indeed established (Bruno and Bertamini, 2013, but see also Suitner and Maass, 2007), our intention is – by no means – to equate selfies with self-portraits. We focus on a complex, yet understudied relationship between selfie posting and creative behaviors in their mundane forms. Instead of asking about van Gogh’s selfies, we explore the direction and strength of the link between creative activity and achievement on the one hand and the intensity of selfie posting on the other. What are the theoretical connections – if any – between creativity and selfying that make any empirical links plausible at all? Should we consider taking and posting selfies as even a potentially creative behavior, or quite the opposite, as a proof of algorithmic, repetitive, and unoriginal activity characterized by a low level of social value? We explore these questions further in this introduction. First, however, we briefly review the state of the art in selfies research, specifically highlighting the findings that informed our inquiry. Next, we present the benefits of analyzing selfies as a situated phenomenon and the need of ecological momentary assessment (EMA) in selfies research.

## Who is Selfying and Why: Knowns and Unknowns

To paraphrase Hermann Ebbinghaus’ famous saying, selfying has a long past, but only a short history. The first selfie was likely taken in 1840 and is attributed to Robert Cornelius: an American amateur photographer and Charles Wheatstone: an English inventor. However, only recently selfies have gathered popularity thanks to the growing availability of smartphones with the reversed camera option (Dhir et al., 2016). Consequently, also scholarly works on selfies flourished only in the last decade searching for predictors of selfie posting (Sorokowski et al., 2015) or demographic differences between people posting more or fewer selfies (Dhir et al., 2016).

Although selfies are becoming more and more popular among social media users, it does not mean they are common. Quite the opposite: a recent summary of the *selfiecity project*^1^ (Tifentale and Manovich, 2016) estimated the number of selfies in all social media sites at only 4% of all photos posted. A look at scholarly works on selfies confirms these estimations; indeed, selfies’ distribution is usually very skewed, with a huge majority of users declaring taking none or only a few selfies, and a clear minority selfying intensively. Such distribution forms an analytical challenge, as typical regression or correlation-based techniques are not robust enough to deal with non-normal distribution. Poisson models or log-transformations are required to handle such a pattern effectively.

Previous studies bring a list of well-corroborated findings regarding selfies. Not surprisingly, selfies were found to be more typical for younger than older social media users (Dhir et al., 2016). Females post more selfies than males (Dhir et al., 2016), although this effect seems to be moderated by selfie type: while selfies that present only a single person are indeed more common among women (Sorokowska et al., 2016), in the case of selfies with a partner or friends, differences are less profound.

What are the personality predictors of selfie posting? Previous studies established a number of selfies’ correlates, but usually these links are, at best, mediocre. Among big five personality factors (McCrae and Costa, 1997) only extraversion seems to be related to selfying, but the effect size of this link is weak (Sorokowska et al., 2016). Indeed, previous studies had found extraversion to be related to general Facebook activity, which, in turn predicts posting photos (Correa et al., 2010; Gosling et al., 2011). The remaining Big Five traits, i.e., agreeableness, neuroticism, conscientiousness, and openness to experience seem to be unrelated to selfying, although future studies would benefit from a more facet-or-aspect-level analysis of personality in this respect (DeYoung et al., 2007).

Previous studies have also examined a range of other personality-related characteristics as selfying’s predictors. For instance, it was hypothesized that self-esteem may influence the intensity of selfie-posting, yet previous studies bring equivocal findings – while some researchers (Nadkarni and Hofmann, 2012) posit that higher self-esteem should translate into more intensive presentation in social media, others demonstrate that lowered self-esteem leads to a more intensive social media practices (Mehdizadeh, 2010). A recent large investigation (Sorokowska et al., 2016) found self-esteem to be unrelated to selfie posting – the one and only positive relationship (*r* = 0.19) was observed among males whose self-esteem was correlated with own selfie posting – a relationship between posting group selfies or selfies with a partner was unrelated to self-esteem in either males or females.

Likely, the most promising line of research in the selfie literature focuses on the dark personality characteristics as its predictors. These studies include exhibitionism (Sorokowska et al., 2016), histrionic personality (Sorokowski et al., 2016), or narcissism (Sorokowski et al., 2015). Consistently with the predictions, in several studies people who posted selfies were found to be higher in narcissism, exhibitionism, and histrionic personality. Two things, however, are important to note. First, the effect size of the links observed was usually tiny. A large sample size made these correlations or regression coefficients statistically significant, however, with coefficients in their 20s there is a lot of room for exceptions. Second, the positive links between narcissism-related traits and selfying were much more consistent among men than women. Hence, paradoxically, although females are more intensive selfie takers and posters, we know less about the causes of their selfying.

Finally, almost all recent studies on selfies have utilized cross-sectional designs (see Guazzini et al., 2016, for an exception). Participants are usually asked how many selfies they posted on social media or how intensively they are *usually* selfying. We believe, however, that the most promising research strategy is to analyze selfies as a *situated phenomenon*. Are people selfying because they are narcissistic or because they are in a place and a moment that they would like to share with their friends? Or perhaps both? Aren’t situational factors – moment-to-moment or day-to-day activity – at least equally as important in explaining the phenomenon of selfie posting? In the study, we present below, we decided to explore this opportunity and focus on selfies as a changeable phenomenon that differs from day to day. Among potential daily predictors of selfying, we see the role played by creative activity. Yet, why and how can creativity be related to selfies? We discuss this issue below.

## (UN)Creative Selfies?

Creativity is understood as a human capacity that allows people to produce ideas and artifacts that are both novel and appropriate. Although creativity scholars often omit the explicit definition of their main construct of interest (Plucker et al., 2004; Silvia, 2014), two aforementioned elements: novelty (originality) and value (usefulness, quality) are perceived as critical definitional criteria for creativity: ingredients of the so-called standard definition of creativity (Runco and Jaeger, 2012). Several additional criteria of creativity were proposed, including surprisingness (Simonton, 2012), esthetics and authenticity (Kharkhurin, 2014); potential (Corazza, 2016); or – long before – transformational power and condensation of meaning (Jackson and Messick, 1965). The last six decades of research within cognitive and personality psychology have also established several traits predictive of creative thinking and problem solving. It was demonstrated that intelligence forms an important, perhaps even necessary, yet not sufficient condition of creativity (Silvia, 2015; Karwowski et al., 2016). Similarly, the role of certain personality traits, mainly openness (Feist, 1998), but also psychoticism (Eysenck, 1995; Acar and Runco, 2012) has been replicated by different labs. Regarding motivational characteristics, there is a widely held consensus that intrinsic motivation is fruitful for creativity (Amabile, 1996), although rewards and extrinsic influences may be conducive to creative thinking as well (Eisenberger et al., 1998; Byron and Khazanchi, 2012). The role of creative self-efficacy – or more generally – creative self-beliefs, has been demonstrated as well, showing how creative self-efficacy, creative personal identity (Jaussi et al., 2007), creative metacognition (Kaufman and Beghetto, 2013), and creative mindsets (Karwowski, 2014; Karwowski and Brzeski, 2017) explain a significant portion of variability in creative efforts, activities, and achievements. *Last but not least*, two distinctions are relevant for understanding creativity. The first distinguishes between creative potential and creative activity or achievement. Creative potential is a complex and multifaceted category of cognitive processes and personality, including divergent thinking (Baer, 2014), creative imagination (Dziedziewicz and Karwowski, 2015), openness to experience (Silvia et al., 2014), or curiosity (Karwowski, 2012). Creative activity denotes time and effort put into different domains: be it science, art or everyday creativity. Finally, creative achievement denotes observable and socially recognized accomplishments – published poems, received patents or awards – all the way to the Pulitzer or Noble Prize. Another relevant distinction differentiates levels of creativity: a personal insight typical for *mini-c creativity*, via *little-c* creative solutions important for everyday problem solving, to *Pro-c* – creative activity in professional activity, and all the way to *Big-C creativity*: eminent form of creative achievements (Kaufman and Beghetto, 2009).

How could creativity and selfying be related? One line of reasoning would put narcissism as a bridge between them. As we already highlighted, narcissism predicts selfying, at least among men. But although the links between creativity and narcissism have been hypothesized for decades (Raskin, 1980), empirical evidence is, at best, equivocal. While some studies demonstrated consistent and robust links between narcissism and self-reported creativity (Furnham et al., 2013; Jonason et al., 2015; McKay et al., 2016), the relationship between narcissism and divergent thinking, creative problem solving (Goncalo et al., 2010), or creative achievement (Jonason et al., 2015) is weaker and less consistent. Therefore, although the relationships “creativity-narcissism” and “narcissism-selfying” may lead to expecting associations between creativity and selfying as well, this rationale is not void of problems.

Covariance of creativity and selfying is also easily inferred from a long tradition of studies that utilize the autophotographic methodology. Autophotography, described in the writings of Ziller and Lewis (1981), Ziller and Rorer (1985) and Ziller (1990/2000), asks participants to take a set of photos that describe their identity and respond to the question “who are you”? Crucially for our argument here, in dozens of investigations Dollinger et al. (1996) and Dollinger and Dollinger (2003) demonstrated fruitfulness of autophotography for studying creativity. They convincingly profiled less creative individuals as those who portray themselves in one-dimensional ways, while observing that more creative people’s photo-essays are not only different, but also much more integrated (Dollinger et al., 1996; Dollinger and Dollinger, 2003). Dollinger described these more metaphorical and esthetically sensitive photo-essays as *individualistic*, and found consistent correlations between individuality and creativity. It is important to note, however, that for Dollinger a selfie is an antonym rather than synonym of highly individualistic photo-essays. As Dollinger (2017, p. 347) put it: “If selfies are included in photo essays—selfies as they are usually portrayed in the media—they would likely result in a low score on individuality/richness.” Although indeed, typical selfies seem to be more imitative and algorithmic than metaphorical and esthetically appealing, this claim is yet to be empirically examined. This is not exactly our aim here, however: as we have mentioned above, it is not our goal to analyze selfies’ content. Instead, we explore the dynamic links between day-to-day creative behaviors and selfying.

## The Present Study

To estimate the level and factors that stand behind intra-individual variability in selfying, we decided to conduct a diary study instead of running the most common cross-sectional studies. Such microlongitudinal approach allows for including within-person predictors such as day-to-day activity as well as several between-person variables, i.e., cognitive abilities, creative achievement or demographic controls. We are primarily interested in the scope of day-to-day variability in selfying, but also in the role played by day-to-day creative activity in different domains and previous creative achievement (measured as a between-person variable) for selfying. We hypothesize that creative activity in art-related domains, and – especially – an activity typical for everyday behavior in spheres related to social media – like blogging or taking photos, will predict the intensity of selfie posting.

## Materials and Methods

### Participants

A total of 292 Polish adults (174 women), aged between 18 and 50: *M*_age_ = 32.77; *SD*_age_ = 8.72) participated in this 2-weeks diary study. All participants were recruited from a larger cross-sectional study (*N* = 803) in which between-level variables: intelligence and creative achievement were measured (about 2 months before the diary study). In the current investigation, we only use data from those of our participants who kept the diary active for no less than a week out of 14 days (*M* = 11.68 days, *SD* = 1.43, range 7–14 days) and were active Facebook users, i.e., declared using Facebook on these days with at least minimal activity every day. The participants were members of an online research panel led by the Millward Brown Poland research company (including close to 100,000 Poles – a representative nationwide sample of Internet users) whose members take part in various research programs once or twice per year. Participants received remuneration for their participation in the form of a voucher valued at 100 PLN (∼25 euro).

### Measures

#### Between-Person Measures

##### Intelligence

To measure intelligence, we selected 30 items developed within the International Cognitive Ability Resource Project (IPAR; Condon and Revelle, 2014). There were ten matrix reasoning items, 10 mental rotations items, seven letter series items, and three overall reasoning items. Reliability of the overall score was good (α = 0.87).

##### Creative achievement

To quantify the level of participants’ previous creative achievement we used Creative Achievement Questionnaire (CAQ; Carson et al., 2005). CAQ measures creative activity in 10 domains: (a) visual arts; (b) music; (c) dance; (d) architecture; (e) writing; (f) humor; (g) inventions; (h) science; (i) theater and film; and (j) kitchen. The total score was skewed (*M* = 6.28, *SD* = 7.35, skewness = 2.26, kurtosis = 5.69), which is typical for CAQ distribution (Carson et al., 2005; Silvia et al., 2012). Therefore, we log-transformed it (skewness = 0.16, kurtosis = -0.38) for multivariate analyses.

##### Between-person controls

We controlled for participants’ age and gender.

#### Within-Person Measures

##### Creative activity

Each day, participants rated the intensity of their engagement in 15 different activities, using a 7-point Likert scale (1 = *not at all*, 7 = *very intensively*).

##### Selfying

Participants rated the intensity of selfie posting during the day, using a 7-point Likert scale (1 = *not at all*, 7 = *very intensively*).

##### Within-person controls

We controlled for weekday (versus weekend) and day-to-day variability in Facebook usage.

### Procedure

After responding to the invitation to participate in a study, participants completed the informed consent form. For 2 weeks, they completed an online daily diary accessible between 6:00 p.m. and 11:00 p.m.

## Results

We proceeded with data analysis in three steps. After an initial overview of descriptive statistics and correlations between variables, we reduced the number of within-person variables using confirmatory factor analysis (CFA). Then, we estimated the level of within-person (day-to-day) and between-person variability in selfying as well as its situational and individual predictors.

Descriptive statistics and intercorrelations between Level-2 (between-person) variables are presented in **Table 1**, while **Table 2** shows descriptive statistics for Level-1 (within-person) variables.

**Table 1:** Descriptive statistics and correlations among between-person variables.

		Min	Max	*M*	*SD*	Skew	Kurt	1	2	3	4	5	6	7
1	Selfie	1.00	4.45	1.25	0.52	3.44	13.89	1	0.30**	-0.18**	0.30**	0.24**	-0.04	-0.02
2	Facebook	1.54	6.80	3.04	1.07	0.90	0.63	0.34**	1	-0.12*	-0.01	-0.02	-0.05	-0.20**
3	Intelligence	0.04	1.00	0.49	0.20	0.13	-0.55	-0.25**	-0.12*	1	0.13*	0.20**	-0.11	-0.13*
4	CAQ	0.00	42.00	6.28	7.35	2.26	5.69	0.13*	0.02	0.20**	1	0.88**	-0.07	-0.13*
5	CAQ-log	0.00	3.76	1.59	0.88	0.16	-0.38	0.13*	0.01	0.20**	0.99**	1	-0.06	-0.11
6	Gender	1 (M)	2 (F)	60%F	–	–	–	0.01	-0.04	-0.10	-0.06	-0.06	1	0.06
7	Age	18	50	32.77	8.72	0.35	-0.95	0.01	-0.21**	-0.13*	-0.11	-0.11	0.08	1

*N = 292; Selfie = the aggregated (daily average) number of selfies from diary module aggregated across all days of the diary study; Facebook = the aggregated (averaged) intensity of Facebook usage from diary module; CAQ, Creative Achievement Questionnaire (raw score); CAQlog, log transformed score in Creative Achievement Questionnaire; Gender was coded *1 = male, 2 = female*; Skew, Skewness; Kurt, Kurtosis. Pearson’s *r*s are presented above the diagonal; Spearman’s ρs below diagonal. ^∗^*p* < 0.05; ^∗∗^*p* < 0.01.*

**Table 2:** Descriptive statistics and factor analyses results for within-person variables.

	*M*	*SD*	Art factor	Science factor	Everyday factor
Selfie	1.25	0.78			
Selfie2	13%	–			
Facebook	3.04	1.52			
Designing clothing items	1.09	0.49	0.76		
Creating choreographies/dancing	1.10	0.55	0.69		
Writing, e.g., poetry, short stories, novels, theatrical plays	1.15	0.67	0.61		
Writing press articles (including e.g., columns)	1.13	0.65	0.56		
Designing buildings/interiors	1.17	0.7	0.56		
Composing musical pieces/playing music	1.12	0.59	0.55		
Painting/drawing/sculpting	1.20	0.78	0.54		
Preparing for public speeches/giving public speeches	1.21	0.78	0.49		
Creating websites	1.15	0.69		0.67	
Programing/creating computer programs	1.17	0.74		0.61	
Writing scholarly papers	1.16	0.73		0.51	
Solving technical/scientific problems	1.66	1.35		0.39	
Creating online blog(s) entries	1.23	0.79			0.67
Taking photos/making videos, e.g., with a phone	1.53	1.09			0.44
Cooking based on one’s own recipes	2.10	1.64			0.27

*N = 3356 occasions (occasion = Number of *Days* ×*Number of Participant*). Seven point Likert scale (*1 = not at all, 7 = very intensively*) was used for all variables except for Selfie2, which was binary coded. Selfie = raw variable describing selfying’s intensity across occasions (7-point Likert scale); Selfie2 = dichotomized (*0 = no, 1 = yes*) variable describing selfying.*

Posting selfies (aggregated across all days of the diary study) was positively linked to the intensity of Facebook usage, Pearson’s *r* = 0.30, Spearman’s ρ = 0.34, previous creative achievement (in the case of raw CAQ score *r* = 0.30, ρ = 0.13; in the case of log-transformed CAQ *r* = 0.24; ρ = 0.13) and negatively to the level of intelligence, *r* = -0.18; ρ = -0.25 (all *p*s < 0.05). Although these initial findings are in line with our expectations, they tell us little about intra-individual-day-to-day variability. Therefore, in the next step we focused on within-level analyses.

### Data Reduction

To reduce the number of within-person variables, we factor-analyzed creative activity variables while controlling for the clustered data at hand (days nested within participants) using Mplus 7.11 and maximum likelihood estimator with robust standard errors. A three-factor CFA model with creative activity in art, science, and everyday creativity fit the data well according to usually applied criteria (Hu and Bentler, 1999), such as confirmatory fit index (*CFI*) = 0.945; Tucker Lewis Index (*TLI*) = 0.932, and root mean square error of approximation (*RMSEA*) = 0.019; and 90% CI:0.015,0.022 (see last three columns of **Table 2** for factor loadings).

**FIGURE 1:**
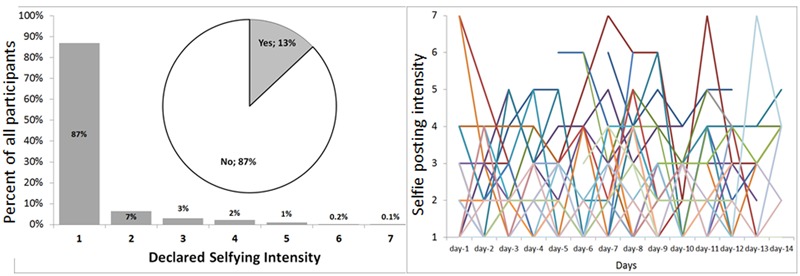
**The distribution (Left)** and day-to-day (within person) variability illustrated on 30 randomly selected participants **(Right)** of selfie posting.

Strikingly, selfie is a rare phenomenon even among Facebook users – only 13% of participants declared posting some selfies during the last 14 days, having an average estimated intensity only slightly higher than 1 (1.25) on a 7-point intensity scale. As expected, selfie posting distribution was skew (**Figure 1**, Left), following a Poisson distribution, similarly as in previous studies (Sorokowski et al., 2016). Creative activity had a similar pattern – among fifteen different activities analyzed, only cooking achieved a mean higher than 2 on a 7-point scale, and some activities – e.g., designing clothing items, creating choreographies, composing music pieces – were almost completely missing (means only slightly higher than 1, with a mode category being *1 = not at all*). Such distribution, however, resembles a pattern that is typical for creative activity (Jauk et al., 2014).

Poisson distribution of the main variables of interests, especially the dependent variable of the intensity of posting selfies informed our decision to proceed with multilevel Poisson regressions. In the first step, however, we estimated an empty multilevel model to obtain the overall level of day-to-day variability in selfying. The intra-class correlation coefficient (ICC) was estimated at 0.36. In other words, 36% of the obtained variability came from between-person differences, while the remaining 64% (1-ICC) was located within person or between days (**Figure 1**, Right). Such a substantial level of intra-individual variability not only justifies our decision to use the multilevel modeling, but is also interesting on its own rights; it demonstrates that selfie posting is to a large extent situation-depended and looking for those aspects of a situation that may cause or predict selfie posting is especially relevant.

The initial multilevel model (**Table 3**, Model A) included five within-person and four between-person predictors. More specifically, we regressed the intensity of selfying on the following within-person variables: the intensity of Facebook usage that day, week-versus-weekend (binary coded), as well as three factors describing creative activity obtained in the CFA: the creative activity in artistic, scholarly, and everyday domains. All these variables (except the dichotomously coded weekend) were group-mean centered around each person’s mean to model changes around each person’s typical behavior across all days. Between-person predictors included two controls: sex and age as well as intelligence and creative achievement. These variables (except the binary coded sex) were grand-mean centered.

**Table 3:** Multilevel models explaining the intensity of selfying.

Predictors	Model A		Model B	
	*B* (*SE*)	Stand Est	*B* (*SE*)	Stand Est
Within-person predictors				
Facebook	0.03 (0.009)	0.29***	0.03 (0.009)	0.29***
Weekend (*0 = no, 1 = yes*)	0.06 (0.022)	0.21**	0.06 (0.022)	0.21**
Artistic creative activity	0.19 (0.039)	0.36***	0.19 (0.04)	0.36***
Everyday creative activity	0.14 (0.023)	0.65***	0.14 (0.023)	0.66***
Scientific creative activity	0.04 (0.026)	0.13	0.045 (0.027)	0.14
Between-level predictors				
Sex (*1 = M, 2 = F*)	-0.05 (0.05)	-0.17	-0.05 (0.05)	-0.17
Age	-0.001 (0.003)	-0.07	-0.002 (0.003)	-0.10
Intelligence (IQ)	-0.51 (0.12)	-0.76***	0.08 (0.17)	0.11
Creative Achievement (CAQ)	0.13 (0.03)	0.81***	0.29 (0.07)	1.76***
*IQ* × *CAQ*	-	–	-0.36 (0.10)	-1.50***

*Bs are unstandardized Poisson regression coefficients with robust standard errors (*SE*) from multilevel modeling that reflect the estimated average within-person relationship for the sample.*

Day-to-day variability in selfie posting has been positively linked to the overall intensity of Facebook usage. Selfies were also more often posted on weekends than weekdays. Importantly, though, and consistently with our hypotheses, selfying was positively predicted by the engagement in creative activity in arts and everyday creative behavior such as blogging or taking photos. In the case of everyday creative activity, the effect size of these differences was substantial (standardized estimate = 0.65). Sex or age did not differentiate the intensity of selfie posting, but intelligence and previous creative achievement did. Strikingly, their effects were opposite; while we have observed a clear and strong positive effect of previous creative achievement on selfying, the effect of intelligence was negative.

For exploratory purposes, we examined several cross-level interactions, but the effect of art-related and everyday creativity on selfie posting was robust: it held among males and females, older and younger participants, people with higher and lower intelligence, and participants with or without previous creative achievements. We have also tested the between-person interaction of *Creative Achievement* × *Intelligence* (**Table 3**, Model B). This interaction was indeed statistically significant.

**FIGURE 2:**
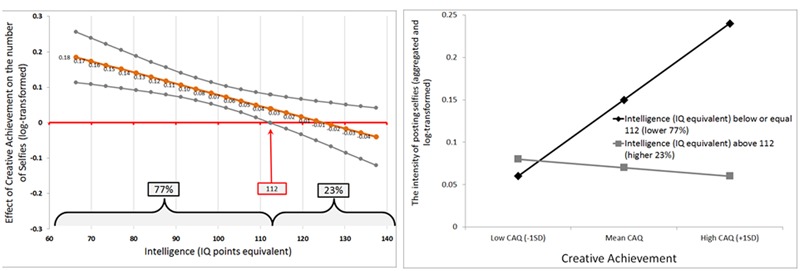
**The relationship between creative achievement and selfying as moderated by intelligence: estimated zone of significant moderation **(Left)** and slopes for people differing in intelligence (Right)**.

Using the Hayes (2013) *process*, we explored this interaction further^2^. More specifically, although the direction of the interaction term suggested that the positive effect of creative achievement on selfie posting may be more profound among less intelligent participants, the Johnson-Neyman technique (Hayes, 2013) has demonstrated that the positive link between previous creative achievement and selfie posting was observed among those 77% of participants whose intelligence was almost one standard deviation above mean or lower (an equivalent of 112 points on the IQ scale; see **Figure 2**, Left). Indeed, the observed effect of creative achievement on selfies was significant among individuals with intelligence up-to-almost-one-standard-deviation above the mean, while it disappeared among those whose intelligence level was within the upper 23% (**Figure 2**, Right).

## Discussion

Is selfying really so common as media seem to suggest? Is it driven primarily by selfiers’ psychological characteristics or is it rather situation-dependent? Is selfie posting related to creativity, and if yes, then how? This diary study explored these questions with the aim of looking at the selfies phenomenon from a slightly different angle than previous research did. More specifically, we were interested in selfies’ dynamic and intra-individual rather than between-individual predictors. Although the findings of this study generally replicate those of previous research, at least some of our results may form an extension of this line of inquiry.

Similarly as in previous research^3^ (see Tifentale and Manovich, 2016; also Sorokowski et al., 2016), our study confirms that selfying, especially intensive selfying, is a rare phenomenon. Despite its growing popularity and media attention, a vast majority of social media users do not post selfies at all. Across the 2 weeks of our investigation, only 13% of all participants declared posting selfies, with a clearly skew distribution: even those who selfied, did it once or twice in 2 weeks. That pattern has both methodological and substantial implications. Methodologically, controlling for this severe skewness is necessary to obtain unbiased estimates. Substantially, an extremely small group of intensive selfie-takers forms a challenge for understanding this niche better. Future studies, then, should apply comparative designs focused specifically on intensive selfie takers.

Consistently with our expectations, the day-to-day variability of selfie posting was visibly (in this investigation: two times) higher than its between-person variance. In other words, to understand selfying, we should focus on the dynamic, situational factors rather than (or at least equally to) on stable, psychological characteristics. This finding seems logical; after all, people are often selfying to share their special moments with others or show places they visit. Indeed, we were able to demonstrate that selfying was more profound on weekends than on weekdays and when people spent more time on Facebook. Interestingly, though, daily creative activity within the domain of widely understood art-related activities and especially during everyday creative activities was positively linked to the intensity of selfying. Those who painted, blogged, or composed music posted more selfies the day they engaged in creative activity. Creative activity in science-related spheres was unrelated to selfying. The creativity-selfying association was also visible on a person level – those social media users who had higher creative achievement selfied more than those with little or no achievement. At the same time, however, intelligence negatively linked to selfying and qualified the relationship between creative achievement and selfying – only among people with an IQ-equivalent of up to about one-standard-deviation-above-the-mean was this link significant.

The links we observed obviously require replication and a sound theoretical explanation. Here, we discuss some plausible, even if a bit speculative explanations of obtained associations with the hope to inspire future investigations. More specifically, we see four potential mechanisms that may stand behind the relationships obtained and that should be more thoroughly examined in future studies.

The first argument for the links between creative activity and selfying may stem from previously discussed correlations between narcissism and creativity (Goncalo et al., 2010) and narcissism and selfying (Sorokowski et al., 2015). Although previous studies provided mixed findings regarding the narcissism-creativity association, we believe that this possibility should not be ignored. Creativity requires an authentic rather than hubristic pride (Damian and Robins, 2013), but more narcissistic, hubristic pride may also associate with creativity under certain conditions. For instance, Damian and Robins (2013) showed that in the condition of anger, creativity and hubristic pride were positively related. Therefore, future studies would benefit not only from controlling for participants’ narcissism in creativity-selfying studies, but also from measuring narcissism in a more detailed way, including its facets, to uncover more subtle relationships.

The second potential mechanism and line of future inquiry is related to emotions as factors responsible for – or at least qualifying – the relationship between creativity and selfying. Previous studies, including those based on EMA (Silvia et al., 2014; Conner and Silvia, 2015), demonstrated that everyday creative behavior is linked to positive, active emotions. Such associations were also hypothesized in the theory of everyday creativity (Richards et al., 1988) and are generally widely accepted in the psychology of creativity (Silvia et al., 2014, but see also Baas et al., 2008, 2011). Therefore, one may expect positive emotions to stand behind both creative activity and selfie posting. It should be noted, however, that although the relationship between creativity and positive emotions is likely reciprocal, creativity theories perceive emotions as a cause of creativity rather than vice versa. It is striking, because in the case of selfie posting the opposite direction seems more plausible. Therefore, it is positive emotions that lead to selfie-posting rather than selfie-posting building positive emotions even if such a hypothesis cannot be fully refuted so easily. So future studies, ideally longitudinal or microlongitudinal, may want to examine the extent to which positive emotions mediate the relationship between creativity and selfie posting or whether it is creative activities that mediate the relationship between positive emotions and selfying. Eventually, it is obviously possible as well that positive emotions and creativity predict selfie posting independently from each other.

The third possibility may consider selfying as a natural consequence or even epiphenomenon of the higher level of activity caused by openness and extraversion and consequently plasticity: the personality meta-factor they form together (DeYoung, 2006). Previous studies consistently confirmed that openness is critical for creative functioning (Feist, 1998; Puryear et al., 2016), while the role of extraversion is much more prominent in the case of self-reported creativity-relevant characteristics, such as creative self-efficacy (Karwowski and Lebuda, 2016). Therefore, it could be argued that selfying is a sub-product of higher activity and a search for different activities and hobbies (Wolfradt and Pretz, 2001). This expectation, however, is weakened by inconsistent and usually weak correlations between selfying and personality. Although indeed, extraversion does predict selfie posting, openness is usually unrelated to selfying. Again, future researchers may want to include an even wider measurement of openness – not only including its aspects (DeYoung et al., 2007), but also specific types of openness (Kaufman, 2013; Kaufman et al., 2015) that predicted creativity in previous studies. As these types of openness differently predicted creative behavior across domains, we are approaching the final point: the issue of domain-specificity versus domain-generality of creativity.

Thus, the fourth point may highlight the domain-specific relationship between creative functioning and selfying. Indeed, in our study we found a robust relationship between selfying and everyday creativity, significant and weaker links between selfying and art-related creative activity, and virtually no relationship between selfie posting and scientific creativity. At the between-person level, although selfying was positively related to the total score of the CAQ, its relationship with intelligence was negative. It suggests that while some forms and domains of creativity – mainly art-based and everyday – may be positively related to selfie posting, other – science-related – are either unrelated or even negatively related to selfying^4^. Scientists and artists differ in terms of their personality (Feist, 1998); different personality traits predict creative achievement in art and science (Kaufman et al., 2015). Even if selfies themselves are rarely artistically creative, artistic activity was positively related with selfying. Again, this opens an intriguing opportunity for future studies. What makes art-related creativity related to selfie posting? Is it a matter of personality characteristics of people who engage into artistic activity or perhaps selfies are still sometimes creative? This question becomes even more important if we keep in mind the role of such everyday activities as blogging, taking photos, or designing new clothing items for selfie posting. There are good reasons to believe that selfying, together with these activities, may form a specific syndrome of behavior, a more typical one for young people and not void of creative elements.

### Strengths and Limitations

The findings we have presented should be interpreted in light of advantages and limitations of the current investigation. Among its strengths, we see applications of the EMA methodology, i.e., the use of a diary study with several within- and between-person predictors. It allowed us to demonstrate that selfying is situation-dependent and within-person variables should be included in studies’ designs to better understand why, when, and what are people selfying for.

The main weakness of this study may be seen in a relatively straightforward measurement of the main dependent variable, meaning selfie posting. Future studies should include more detailed sets of questions regarding the different types of selfies. Most importantly, however, future investigations should allow for analyzing selfies’ content. Following an interesting take of the selfiecity project^5^ (Tifentale and Manovich, 2016), it would be interesting to see not only whether creative behavior predicts selfie posting, but also to assess the creativity of selfies. Using big data available in social media may make such a research project possible.

## Ethics Statement

This study was carried out in accordance with the recommendations of The Maria Grzegorzewska University with written informed consent from all subjects. All subjects gave informed consent in accordance with the Declaration of Helsinki. The protocol was approved by The Maria Grzegorzewska University Institutional Review Board (decision number 128-2016/2017).

## Author Contributions

MK planned the whole study, performed statistical analyses, and drafted the manuscript. AB co-analyzed the results and provided a critical overview of the manuscript. Both authors read and approved the final version of the manuscript.

## Conflict of Interest Statement

The authors declare that the research was conducted in the absence of any commercial or financial relationships that could be construed as a potential conflict of interest.

## Footnotes

1 selfiecity.net

2 As this cross-product effect was observed between Level-2 (between-person) variables, we decided to aggregate the number of selfies within individuals (sum they up) and conduct these analyses on Level-2 (person) data.

3 selfiecity.net

4 As the total score of the CAQ is aggregated across different domains, we tested our reasoning in a separate regression on aggregated, between-person level data. We regressed selfying on 10 CAQ domains. The model explained 20% of selfies variability and, as hypothesized, significant predictors were those that related to art rather than science. More specifically, selfying was predicted by creative achievements in music (β = 0.21; *p* < 0.001), dance (β = 0.15; *p* = 0.007), architecture (β = 0.19; *p* = 0.003), and theatre (β = 0.18; *p* = 0.006).

5 selfiecity.net
